# ﻿A new species of *Echinodorus* (Alismatales, Alismataceae) from the Chaco region with a waterlily soul

**DOI:** 10.3897/phytokeys.269.170618

**Published:** 2026-01-07

**Authors:** Pablo A. Calviño, Samuli Lehtonen, Marcos Waldbillig, Tomás Acuña, Felipe Alonso

**Affiliations:** 1 Killifish Foundation, La Plata, Provincia de Buenos Aires, Argentina Killifish Foundation La Plata Argentina; 2 Biodiversity Unit, University of Turku, 20014 Turku, Finland University of Turku Turku Finland; 3 Instituto de Investigaciones en Producción Animal (INPA), Universidad de Buenos Aires (CONICET), Buenos Aires, Argentina Universidad de Buenos Aires (CONICET) Buenos Aires Argentina; 4 Universidad Nacional de la Plata (UNLP), La Plata, Provincia de Buenos Aires, Argentina Universidad Nacional de la Plata (UNLP) La Plata Argentina; 5 Instituto de Bio y Geociencias del Noroeste Argentino (IBIGEO), Consejo Nacional de Investigaciones Científicas y Técnicas (CONICET), Facultad de Ciencias Naturales, Universidad Nacional de Salta (UNSa), Salta, Provincia de Salta, Argentina Universidad Nacional de Salta (UNSa) Salta Argentina

**Keywords:** Alismataceae, aquatic plant, Argentina, Chaco region, *
Echinodorus
*, Paraguay

## Abstract

We describe *Echinodorus nenufar*, a new species from ephemeral aquatic environments in the dry Chaco region of northern Argentina and western Paraguay. This species is readily distinguished by its unique floral coloration—pale yellow to creamy white petals with a basal yellow blotch—and its floating leaf blades, which are broadly cordate to suborbicular, evoking the morphology of waterlilies (Nymphaeaceae). The species exhibits exceptional phenotypic plasticity, producing linear, ovate, or rounded leaves depending on its developmental stage and hydrological context. It completes its life cycle in temporary ponds and seasonal wetlands, where multiple life stages may coexist simultaneously. Molecular phylogenetic analyses based on nuclear (ITS, *LEAFY*) and plastid (*rpl22–rps19*) markers confirm the distinctiveness of *E. nenufar* within the *E. grandiflorus* complex. We also introduce a novel protocol for preserving delicate aquatic plant structures for herbarium curation. Due to its aesthetic appeal, adaptability, and ease of cultivation, *E. nenufar* might become a valuable ornamental species.

## ﻿Introduction

Alismataceae is a cosmopolitan family, with its highest diversity concentrated in tropical and subtropical regions (Les and Tippery 2013). Representatives of this family also extend into temperate zones (Rataj 1970; Novara 2008). The family currently comprises 17 genera and ca. 113 species of aquatic or semi-aquatic plants, according to Li et al. (2022) (Les and Tippery 2013; Lehtonen 2018). Several species are of economic significance, particularly as ornamental plants for aquaria and aquatic landscaping (Kasselmann 2003; Lehtonen and Rodríguez-Arévalo 2005).

In the Chaco region of Argentina and Paraguay, Alismataceae is represented by five genera: *Echinodorus* Rich. & Engelm. ex A. Gray, *Helanthium* (Benth. and Hook.f.) Engelm. ex J.G. Sm., *Hydrocleys* Rich., *Limnocharis* Bonpl., and *Sagittaria* L. The genus *Echinodorus* is restricted to the American continent and currently comprises approximately 28 valid species (Lehtonen 2008). Ten species are recorded for Argentina and Paraguay according to Zuloaga et al. (2025): *Echinodorus berteroi* (Spreng.) Fassett, *E. longipetalus* Micheli, *E. paniculatus* Micheli, *E. uruguayensis* Arechav., *E. reptilis* Lehtonen, *E. floribundus* Seub. ex Warm., *E. longiscapus* Arechav., *E. grandiflorus* (Cham. and Schltdl.) Micheli, *E. subalatus* (Mart.) Griseb. from Paraguay, and *E. scaber* Rataj, the latter of which was recently added to the Argentine flora by Cantero et al. (2016).

In this study, we describe a previously undocumented species of *Echinodorus* within the *E. grandiflorus* complex from the dry Chaco region of northern Argentina and western Paraguay. In addition to its taxonomic novelty, the work presented here incorporates methodological innovations in botanical curation and the preservation of aquatic plants. Specifically, we 1) describe, illustrate, and preserve the floral, foliar, and root variability of the species; 2) develop a novel protocol for optimal herbarium leaf preservation; 3) utilize newly adapted materials and mounting techniques for improved long-term conservation; and 4) propose a refined method for pressing and drying aquatic plant specimens.

## ﻿Materials and methods

### ﻿Specimen collection and preservation

Specimens were collected from temporary aquatic environments in the western Chaco region and subsequently processed and deposited at the Instituto Darwinion Herbarium (SI). To preserve coloration and delicate structural characters, an optimized protocol was developed. Entire plants or isolated leaves were first rehydrated by immersion for 2 hours in a 1 L aqueous solution containing 5 g of ascorbic acid at ambient temperature (25 °C), functioning as an antioxidant to prevent pigment oxidation and cellular collapse.

Following pre-treatment, floral structures were disarticulated and dried individually. Each flower was gently flattened between sheets of absorbent paper and covered with a transparent polypropylene layer to maintain its arrangement and prevent sticking to the recipient material. The prepared specimens were then placed in airtight containers saturated with silica gel and left for 24 hours, allowing complete desiccation while preserving their natural morphology.

Leaves or entire plants were removed from the ascorbic acid solution and pressed, arranged between acid-free white Canson XL Aquarelle watercolor sheets (A3, 300 g/m^2^, ISO 9706, with anti-fungal treatment), and interleaved with several sheets of newspaper until achieving a minimum stack thickness of 5 mm. If necessary, newspaper and watercolor sheets were replaced every 12 to 24 hours. The press was constructed using rigid wooden boards (40 × 30 × 2 cm) perforated with 108 evenly distributed 10 mm holes to enhance air circulation. The entire assembly was secured with four manual bar clamps and placed over a constant-speed fan operating at room temperature (25 °C) for 7 days, with daily inspections.

Mounted specimens were prepared using Canson XL Aquarelle sheets as the base and protective cover. Structural elements (petioles, laminae, roots, and floral components) were secured using transparent polypropylene tabs affixed with white OPP polypropylene archival labels. Final assemblies were housed in A3 polypropylene sleeves, left partially open to enable air exchange. This system is intended to allow moisture regulation and further chemical stabilization, with excess humidity absorbed by the Canson paper layers to ensure long-term specimen integrity and to prevent microbial growth.

### ﻿DNA extraction and sequencing

DNA samples were processed following a modified version of the teabag drying method described by Wilkie et al. (2013). Healthy, young leaves or shoots were cut into 1 cm segments, placed in empty tea bags, and rapidly dried within airtight containers containing non-toxic silica gel (2–4 mm, white and orange spheres).

Total genomic DNA was extracted using the NucleoSpin Plant II kit (Macherey-Nagel, Germany). Three genomic regions were amplified via polymerase chain reaction (PCR): the nuclear ribosomal internal transcribed spacer (ITS) region using primers ITS5 (White et al. 1990) and ITS-ER (Lehtonen and Myllys 2008); the second intron of the nuclear *LEAFY* gene using primers LFY_Flint2-R1 and LFY_Flint2-F1 (Grob et al. 2004); and the plastid intergenic spacer *rpl22–rps19* using newly designed primers 89872F (CGC CCG CGA ATT TGA TCA AT) and 90794R (TGG GAG AAT TCG TAC CTA CTC G). The latter primer pair was developed using Primer3 (Untergasser et al. 2012), based on the plastome of *Echinodorus grisebachii* Small (GenBank accession OR999317). PCR products were sequenced by Macrogen Inc. (Seoul, South Korea/Amsterdam, Netherlands).

### ﻿Allele phasing and sequence alignment

In four individuals of *E. nenufar* and one of *E. longiscapus*, overlapping chromatogram signals were observed in the *LEAFY* sequences, indicating heterozygosity. In these samples, the alleles were phased manually by comparing bidirectional reads and resolving signal overlap, particularly in a 7 bp insertion region. One *E. nenufar* specimen containing only the shorter allele served as a reference to resolve the phased variants in the heterozygous samples.

All sequence alignments were conducted using MAFFT v.7.453 (Katoh and Standley 2013). In heterozygous samples, the shorter *LEAFY* allele was concatenated with the ITS and *rpl22–rps19* sequences for the combined analysis, as it was present in all samples of *E. nenufar*, while the longer alleles from these samples were added alone to the final combined data matrix. Phylogenetic analyses were carried out separately for each locus and for a concatenated dataset with phased alleles using TNT v.1.6 (Goloboff and Morales 2023), employing the search commands mult = tbr replic 1000 hold 10; bbreak = tbr. Support values were calculated through 1,000 jackknife replicates under the same heuristic parameters used for full-tree searches. Furthermore, a Bayesian tree was inferred for the concatenated dataset using MrBayes v3.2.7a (Ronquist et al. 2012). Optimal substitution models for each marker were identified using ModelTest-NG (Flouri et al. 2014; Darriba et al. 2020). The models HKY+I, GTR, and K80+G were selected for ITS, *rpl22–rps19*, and *LEAFY*, respectively. Two runs with four chains of 10,000,000 generations each, sampling every 2000 generations, were completed. The standard deviation of split frequencies was below 0.01, and effective sample sizes (ESS) for each estimated parameter were well over 200. The first 25% of the trees were discarded as burn-in, and the remaining trees were summarized (Fig. 7).

### ﻿Morphological and comparative analysis

Specimens cited in Lehtonen (2008) were reviewed across multiple herbaria, taking into account ecological, phenological, morphological, and biogeographic variation. The species description followed classical taxonomic methodology and was supplemented with detailed illustrations (ink drawings), in situ and ex situ photographs, and a full range of morphological observations from juvenile to reproductive stages. Leaf measurements included full blade length, which, in mature individuals, exceeds the linear distance from the apex to the petiole insertion due to the presence of overlapping basal lobes. Phenology and floral ontogeny were observed under laboratory cultivation in 16 plants, each of which produced a minimum total of approximately 20 flowers from December to April.

## ﻿Results

### ﻿Taxonomic treatment

#### ﻿ Echinodorus nenufar

Taxon classificationPlantaeAlismatalesAlismataceae

P.A.Calviño, Lehtonen, M.Waldbillig, T.Acuña & F.Alonso sp. nov.

59BF0A8C-BF69-5B1C-BA0B-F08EB09D8E93

urn:lsid:ipni.org:names: 77368344-1

Fig. 1

##### Type.

**Argentina** • **Salta**: Departamento Gral. José de San Martín, temporary aquatic habitats along Route 53 near Padre Lozano (23°13'00.06"S, 63°47'55.95"W), 22 November 2024, fl., fr., P.A. Calviño and M. Waldbillig s.n. (holotype: mounted on A-B sheets SI-226624, sheet A, barcode 147436; sheet B, barcode 147435; Fig. 2A, B. Isotype: SI-226624, sheet C, barcode 147423).

##### Diagnosis.

*Echinodorus nenufar* is readily distinguishable from all other members of the genus by its unique floral coloration—petals ranging from pale yellow to creamy white (versus consistently intense white in other species)—and the shape of its mature floating leaf blades, which are broadly cordate to ovate to suborbicular (versus typically ovoid, lanceolate, elliptic, or oval in congeners).

##### Description.

Herbs perennial or annual, rooted hydrophytes, either emergent or submerged. Rhizomes short, fusiform, erect, firm, 10–30 mm long, 15–25 mm in diameter, with fasciculate, fibrous roots, some of which are finely septate and spongy.

Leaves submerged, with floating blades or emergent; venation campylodromous. In mature plants, emergent aerial blades ovate to oval, 9–16(–19) cm long × 8–12(–16) cm wide, with 10–13 veins, green to light green, lacking pellucid lines, apex obtuse to rounded, base cordate, often with basal lobes contiguous or overlapping to form an oval; petioles sheathing at base; epidermis with faint longitudinal striations.

Juvenile emergent leaves. Blades ovate, 3–5 × 2–3 cm, with 5–7 veins and a cordate base. Floating leaves. Blades ovate to broadly ovate or suborbicular, with a cordate base whose basal lobes often overlap; (2.5–)7–15(–26) cm long × (1.5–)4–12(–18.5) cm wide; lacking translucent markings. Petioles from 10 up to 50 cm long and 2–8 mm in diameter, with 7–11 veins and indumentum composed of glandular, papillate trichomes bearing 1–3 dendritic or stellate hairs. Submerged leaves. Blades initially linear-lanceolate, later lanceolate, with attenuate bases, obtuse apices, and 3–5 veins. Petioles triangular in cross-section, winged at the base; early petioles subpetiolate, later ones up to 20 cm long and 5–10 mm in diameter.

**Figure 1.**
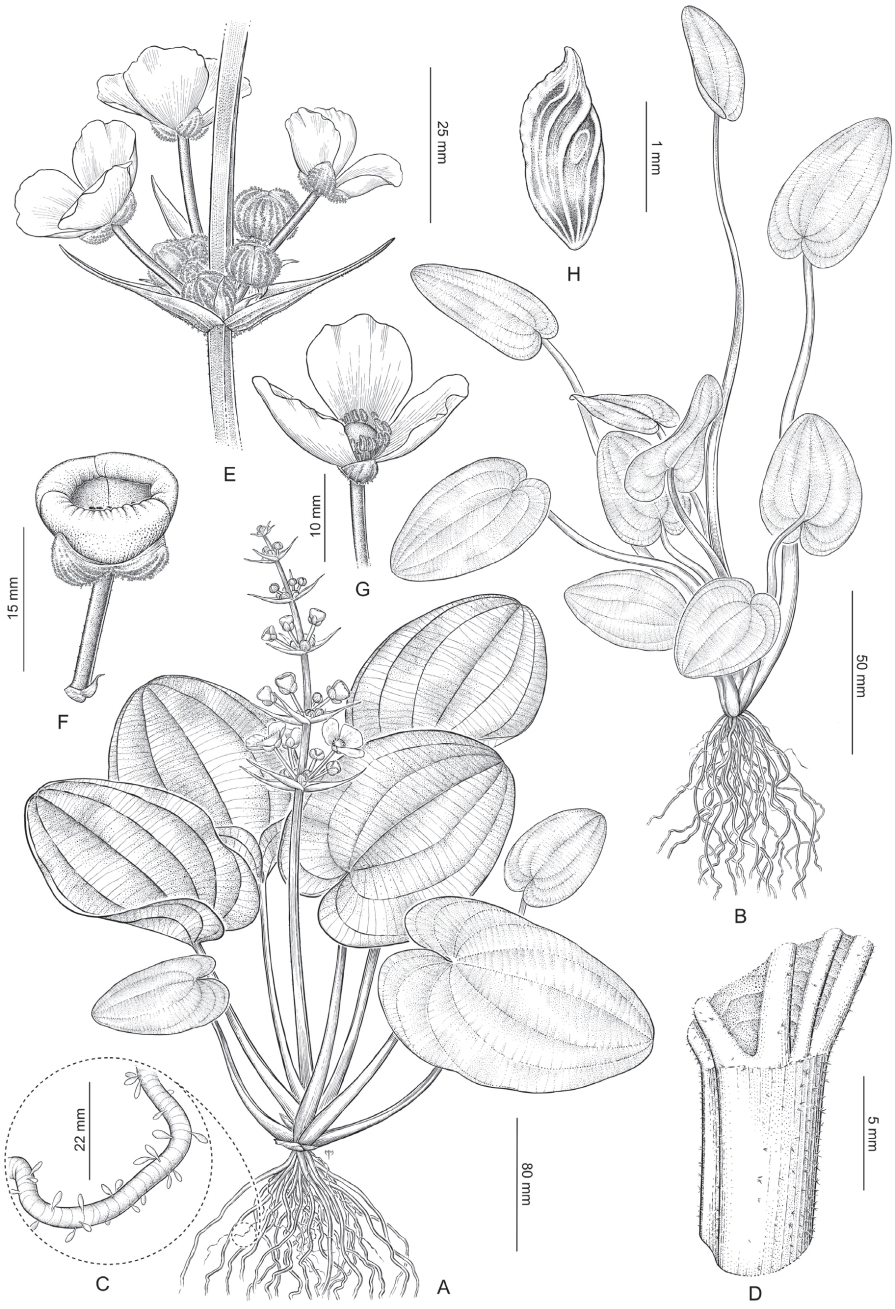
*Echinodorus nenufar*. **A.** Mature plant; **B.** Juvenile submerged plant; **C.** Detail of root with finely ringed velamen and terminal drop-shaped trichomes; **D.** Petiole apex with trichomes; **E.** Floral whorl; **F.** Bowl-shaped flower at ~ 4:00 PM; **G.** Cup-shaped flower with erect petals by midday; **H.** Achene with dorsal keel and basal gland. Illustration by Marcelo Moreno.

Juvenile emergent leaves ovate, 3–5 cm × 2–3 cm, with 5–7 veins and cordate base. Floating blades ovate to broadly ovate or suborbicular, base cordate with often overlapping basal lobes, (2.5–)7–15(–26) cm long × (1.5–)4–12(–18.5) cm wide, lacking translucent markings, petioles up to 50 cm long and 2–8 mm in diameter, with 7–11 veins and indumentum composed of glandular, papillate trichomes, bearing 1–3 dendritic or stellate hairs. Submerged blades initially linear-lanceolate, then lanceolate, with attenuate bases and obtuse apices, 3–5 veins, petioles triangular in cross-section, winged at base, early leaves subpteriolate (with a very short or partially differentiated petiole, intermediate between sessile and fully petiolate); later ones up to 20 cm long, 5–10 mm in diameter.

**Figure 2.**
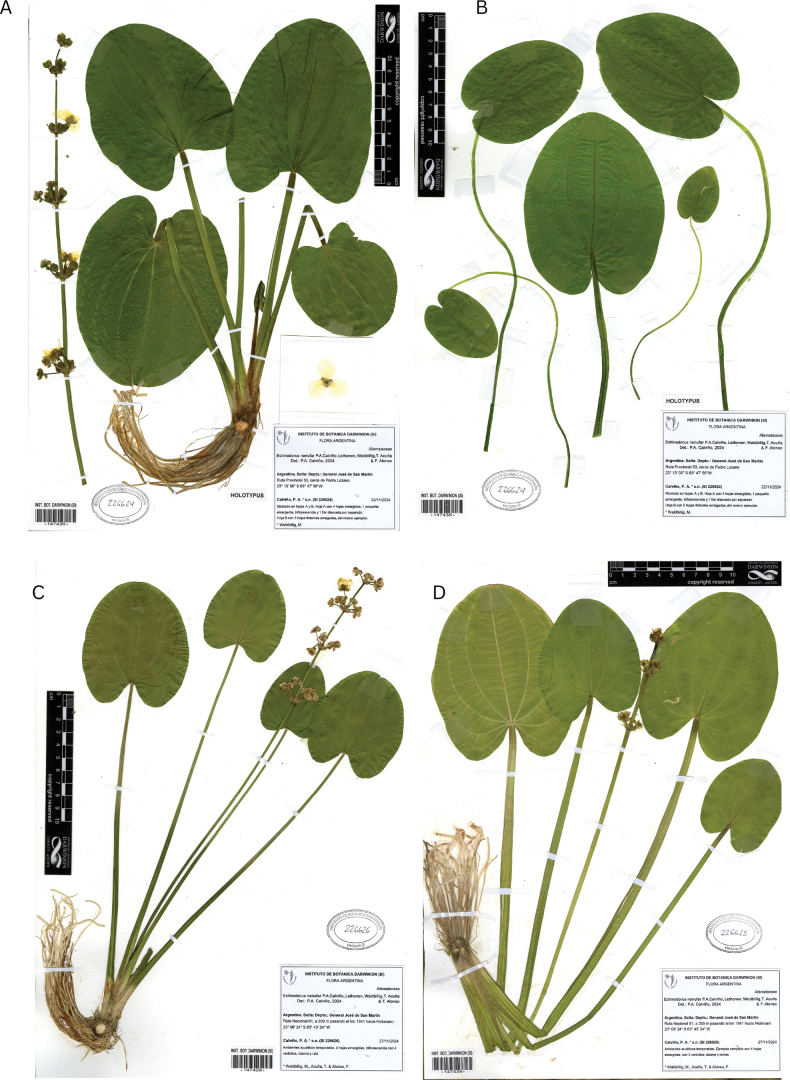
*Echinodorus nenufar*. **A, B.** Holotype mounted on two sheets (SI 226624, sheet A barcode 147436, sheet B barcode 147435); **C.** Paratype (SI 226626, barcode 147428); **D.** Paratype (SI 226625, barcode 147434).

Inflorescences racemose or paniculate, bearing 4–7 whorls of flowers, each whorl with 3–14 flowers, upright or decumbent, often proliferous, up to 45 cm tall and 10 cm wide. Rachis triangular in cross-section, longitudinally striated, puberulent, with some glandular and dendritic trichomes. Peduncles terete, up to 50 cm long and 7.5 mm in diameter, sparsely pilous. Bracts lanceolate, robust, 1.5–4.5 cm long, sometimes shorter, equal to or longer than subtended pedicels, with 10–17 veins, apex acute. Pedicels cylindrical, terete, ascending, accrescent to 0.5–3.5 cm long, 1.0–1.5 mm in diameter.

Flowers bisexual, 1.5–2.5 cm in diameter, ephemeral, each lasting a single day. Sepals three, green, erect, ovate, obtuse, concave, 10–14-nerved, ca. 5 × 3 mm, abaxially bearing whitish to hyaline trichomes, filiform, branched, or dendritic. Petals three, obovate to orbicular, delicate, non-overlapping to slightly imbricate, pale yellow to creamy white, often with a deeper yellow basal blotch extending up to one-third of the petal length, ca. 12 × 13 mm. Stamens 19–26; filaments slender, ca. 3.5 mm long; anthers oblong, subbasifixed, versatile, ca. 3 mm long. Gynoecium apocarpous; style simple; carpels numerous. Fruits achenes, oblanceolate, ca. 1.5 × 0.5 mm, with 4–6 lateral ribs and 2–4 dorsal keels extending toward the persistent style beak, usually bearing 1–3 small irregular glands; style beak 0.2–0.4 mm long, erect, or lateral.

Floral morphology and pigmentation are markedly variable. Most individuals display the typical cup-shaped corolla with erect petals, pale yellow to creamy white, each marked by a conspicuous yellow basal spot that fades gradually toward the center. A smaller proportion shows distinct morphotypes. In one variant, petals are shorter and uniformly deep yellow yet retain the cup-shaped configuration. Another, less frequent form, has fully expanded, non-overlapping, creamy-white petals with a pale yellow to lime-green basal blotch; rare individuals present slightly overlapping petals of similar hue and pattern.

**Figure 3.**
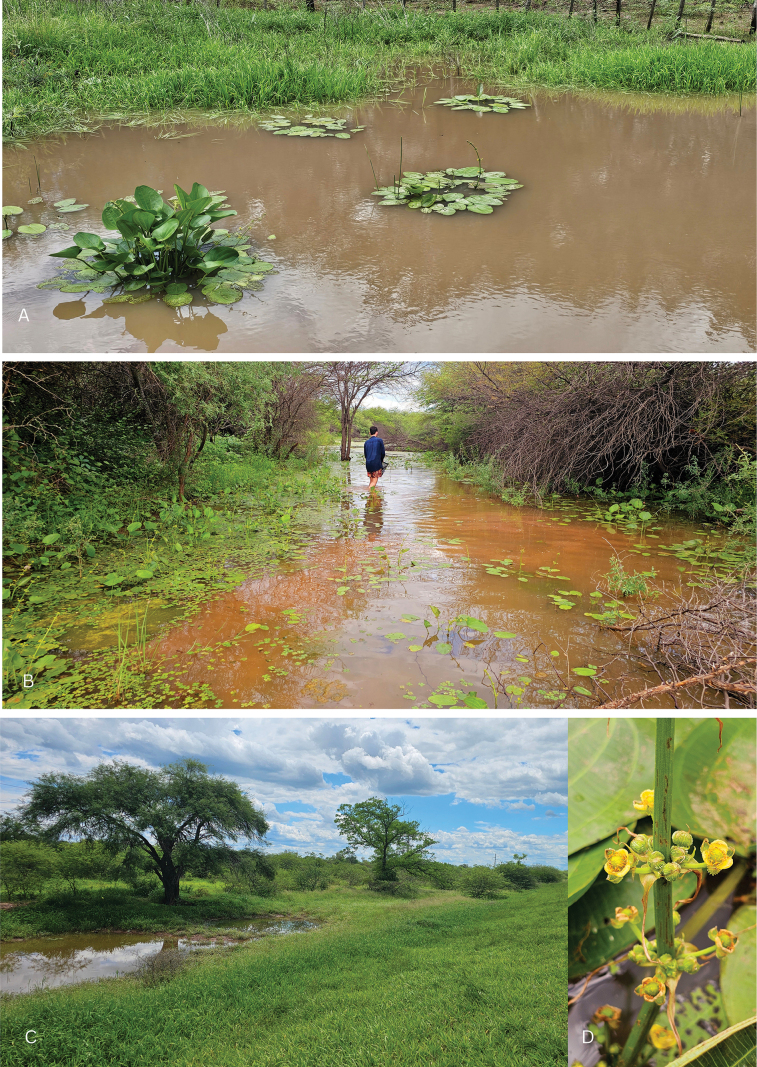
Habitat views of *Echinodorus nenufar*. **A.** Ephemeral wetland near Route 81, close to Hickmann (–23.173501°S, –63.662227°W), 22 November 2024; **B.** Type locality; **C.** Same environment as (**A**); **D.** Inflorescence detail at dusk. Photographs and plate design by P.A. Calviño.

##### Morphological variation.

**Figure 4.**
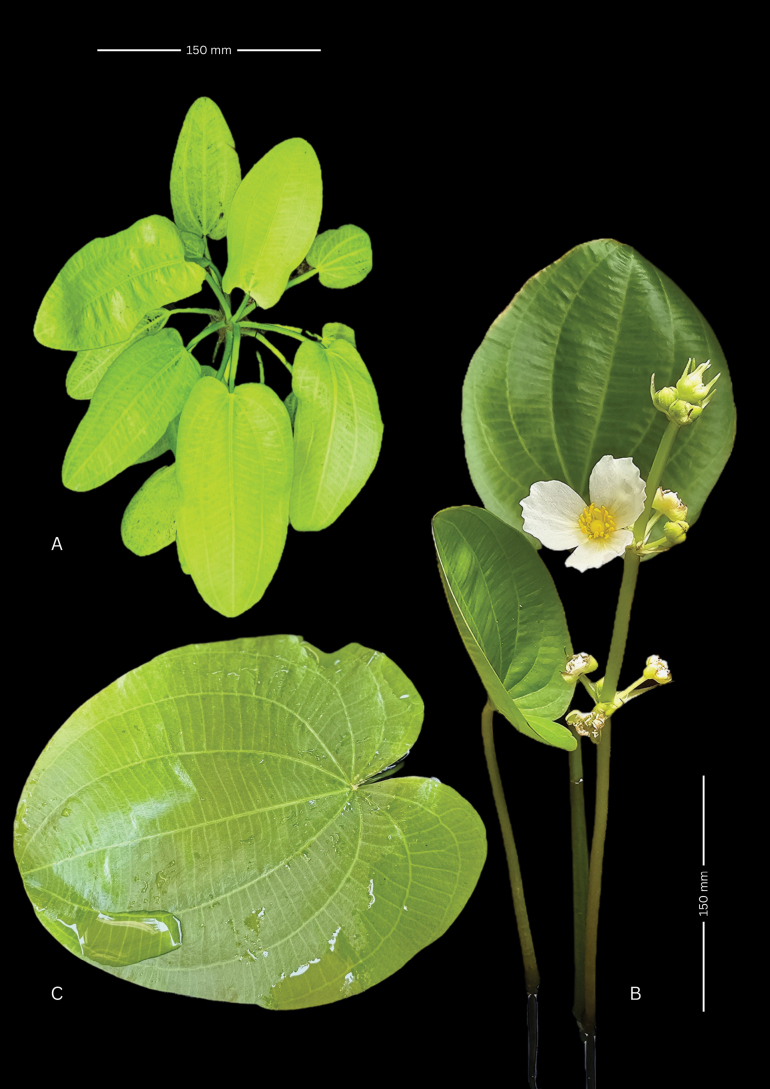
Growth habits of *Echinodorus nenufar*. **A.** Submerged habit, top view; **B.** Emergent habit; **C.** Floating leaf blade; **A.** From SI 226627; **B.** From SI 226625; **C.** From SI 226630. Photographs and plate design by P.A. Calviño.

*Echinodorus nenufar* exhibits pronounced morphological plasticity throughout its life cycle, thriving under both terrestrial and aquatic conditions. Germination and early development may occur either submerged or on exposed, periodically inundated soil. The highest degree of heterophylly is expressed in seedlings developing underwater. In submerged seedlings, the first two leaves are linear-lanceolate, followed by a pair of lanceolate blades; the fifth and sixth leaves become ovate (Fig. 4A), and later growth gives rise to cordate-ovate floating leaves (Fig. 4C). Mature individuals in aquatic habitats produce broadly ovate to suborbicular, cordate floating blades. At this stage, the root system remains fibrous, and petioles are initially short.

In fully submerged plants, the leaf blades float at the surface, borne on elongated petioles that connect them to the rhizome. These petioles, reaching up to 55 cm in length, are slender and triangular to subcircular in cross-section, with a shield-shaped base. The blades are thin, ovate-cordate to oval-cordate. Developmentally earlier, narrower (lanceolate or elliptical) leaves may remain submerged or reach the surface. Larger floating leaves may become nearly circular, closely resembling those of *Nymphaea* (Nymphaeaceae), particularly before the emergence of aerial inflorescences.

During maturation, individuals transition to an emergent phase characterized by robust ovate to oval, cordate leaves that typically surround the central inflorescence (Fig. 4B). The basal lobes of these leaves may overlap and curve upward, forming a shallow spoon- or bowl-like shape, while older floating leaves persist toward the plant periphery. This phase generally coincides with the late stages of habitat desiccation. Emergent leaves in terrestrial individuals retain a cordate base, and their lobes may overlap to form a nearly perfect oval outline. When lying flat against the substrate, these leaves closely resemble those of *Nymphaea*.

Seedlings germinating in terrestrial microhabitats often persist as dwarf rosettes with ovate, cordate-based leaves, forming dense clusters. These forms may later revert to aquatic morphologies upon flooding. Conversely, the sudden inundation of juvenile terrestrial plants induces the formation of elongated petioles, which raise newly produced leaves to the water surface, initiating the floating phase. Submerged leaves from earlier stages usually retain their shape, while newly developed ovate leaves may remain permanently submerged in some individuals. Trichomes are restricted to aerial parts. Leaf venation is occasionally tinted purple, and entirely purple leaves occur rarely.

Variation in petal coloration and corolla form appears to be environmentally influenced, particularly by light intensity. Flowers with expanded, cream-colored petals are consistently found in plants growing in partial shade beneath trees, whereas cup-shaped, yellow to cream flowers predominate in full-sun habitats. Under cultivation (pers. obs.), an inflorescence collected from a sun-exposed wild plant produced pale cream flowers when grown under shaded aquarium conditions. Yellow pigmentation intensifies during senescence, coinciding with petal curling or retraction at the distal margins preceding wilting. Individual plants usually bear flowers of uniform color and form, suggesting that environmental effects operate at the level of the whole plant rather than on individual flowers.

**Figure 5.**
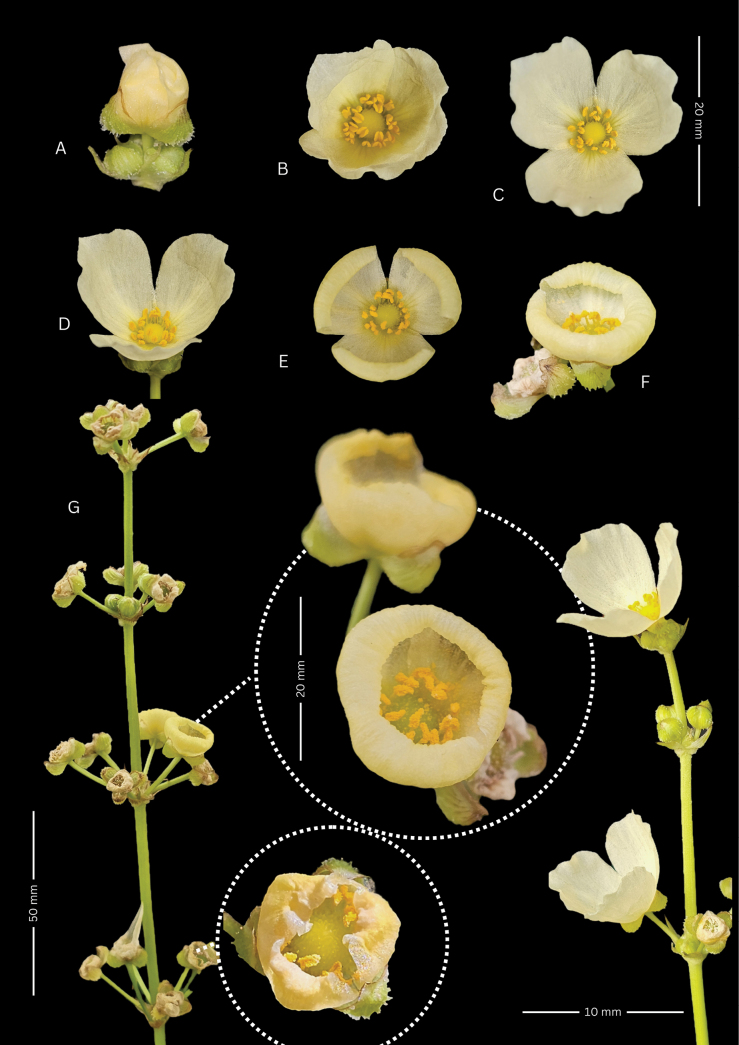
Floral ontogeny of *Echinodorus nenufar*. **A.** Bud emergence at 6:00 AM; **B.** Initial petal unfolding in a rosette-like form at dawn; **C.** Erect, cup-shaped corolla maintained until midday; **D.** Maximum petal expansion under full sun; **E, F.** Onset of petal retraction, forming an open or closed bowl by ~4:00 PM; **G.** Inflorescence rachis detail in late afternoon. Photographs and plate design by P.A. Calviño.

Floral opening follows a consistent diurnal sequence (Fig. 5). Around 05:30 h, sepals begin to separate; by 06:00 h, the floral bud opens in a rosaceous pattern at dawn. At approximately 07:30 h, the petals unfold, and the corolla assumes an erect, cup-like shape. By midday (12:00–13:00 h), the corolla is fully expanded—flowers in full sun maintain a cup shape, while those in shaded conditions may become more expanded or reflexed. Around 14:30 h, petals begin to retract subtly; by approximately 16:00 h, the corolla may form a shallow bowl or close entirely, particularly in flowers that did not fully expand during anthesis. Senescence begins around 17:00 h; petal closure accelerates under direct sunlight but is delayed in shade.

**Figure 6.**
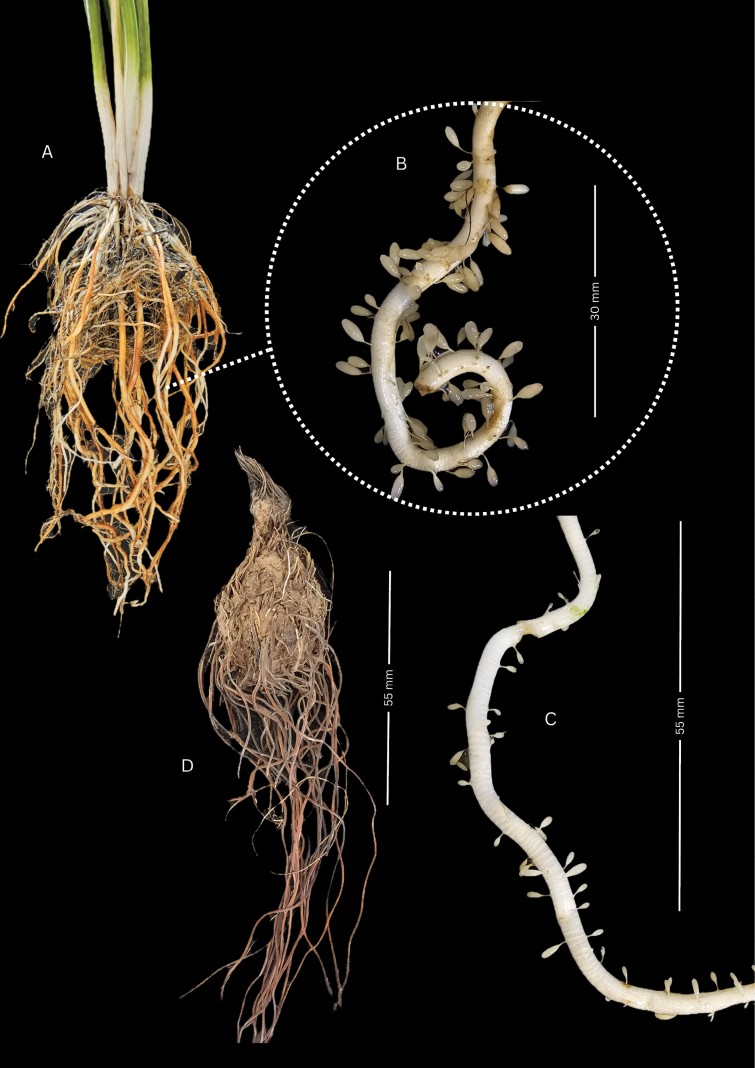
Root morphology. **A.** Thick, fibrous roots with reddish surface coloration due to laterite in the substrate; **B, C.** White, hydrated, fleshy roots with velamen and terminal drop-shaped nodules; **D.** Fusiform rhizome with fibrous roots. Photographs and plate design by P.A. Calviño.

Root structure in *Echinodorus nenufar* is notably variable and includes adaptations to temporary aquatic environments (Fig. 6). In several adult specimens (e.g., SI 226628) cultivated in partially submerged pots, the roots were spongy, white, and septate, surrounded by a velamen-like sheath that envelops and protects the fibrous axis. This velamen is semi-translucent and parchment-like when dry, imparting a pearly white appearance; when hydrated, it becomes gelatinous and flexible, presumably enhancing oxygen diffusion and providing mechanical protection. Specialized root hairs arise laterally through the velamen and terminate in nodular, drop-shaped apices that swell markedly when hydrated, increasing the absorptive surface area. In dried specimens, the velamen often contracts and may detach, leaving the fibrous core exposed; its presence and condition therefore vary depending on environmental conditions and specimen preservation.

##### Distribution and ecology.

*Echinodorus nenufar* is currently known from temporary aquatic habitats in the western Chaco region of northern Argentina (Salta Province) and western Paraguay (Boquerón Department), at elevations of 250–270 m a.s.l. (Fig. 3). Between late November and early May, individuals were observed at a range of developmental stages across various habitats. These stages include submerged, floating, and emergent blades, as well as mature and juvenile terrestrial forms. Among these, floating and juvenile terrestrial forms were most frequently observed, whereas fully mature emergent individuals were relatively uncommon.

The species appears to exhibit a life-history strategy analogous to that of seasonal killifishes (Cyprinodontiformes, Rivulidae) inhabiting the same ephemeral environments (Calviño et al. 2016; Alonso et al. 2018). Like these fishes, *E. nenufar* seems to rely on the simultaneous presence of multiple life stages and overlapping generations as an adaptation to the unpredictability of flooding and drought events beyond typical seasonal cycles.

##### Phenology.

Specimens bearing flowers and fruits were collected in the field from November through May.

##### Conservation status.

*Echinodorus nenufar* is currently known only from a few geographically close localities in the Argentine and Paraguayan Chaco, and its true abundance and full distribution remain uncertain. The species inhabits temporary aquatic environments that undergo extreme fluctuations driven by flood–drought dynamics, conditions likely to produce substantial and unpredictable variation in population size, although a persistent seed bank is probable. The dry Chaco is undergoing rapid agricultural conversion and associated ecosystem degradation (Barral et al. 2020), further increasing risk. Based on the presently known extent of occurrence (EOO) of approximately 420 km^2^ and area of occupancy (AOO) of 12 km^2^, together with presumed population fluctuations and recent habitat decline, we tentatively assign an Endangered (EN) category under criteria B1abc(iii)+2abc(iii) of the IUCN Red List (IUCN 2024). However, if additional populations are discovered—as is considered likely—a Vulnerable (VU) category may be more appropriate.

##### Etymology.

The specific epithet *nenufar* refers to the Spanish common name “nenúfar,” used to designate water lilies (Nymphaeaceae). The name highlights the striking resemblance between the broad floating leaves of *Echinodorus nenufar* and those of true water lilies, particularly in habit and overall architecture.

##### Additional specimens examined (paratypes).

**Argentina** • **Salta**: Departamento Gral. José de San Martín, Ruta Nacional 81, a 200 m pasando el km 1841 hacia Hickmann, 23°08'24"S, 63°43'24"W, 27 Apr 2024, *P.A. Calviño, M. Waldbilling, T. Acuña & F. Alonso s.n.* (SI-226625, barcode 147434!)., Fig. 2D. • Route 53 near Padre Lozano, 23°13'00.06"S, 63°47'55.95"W, elev. 260 m, *s.n.* 28 Apr 2024, fl., fr., *P.A. Calviño and M. Waldbillig s.n.* (SI-226626, barcode 147428!; SI-226627, barcode 147437!); • 22 Nov 2024, fl., *P.A. Calviño s.n.* (SI-226628, barcode 147431!; SI-226629, barcode 147430!). **Paraguay** • **Boquerón**: 11 Km. de la pista 4ta. División de Infanteria, s/ Línea 10. En algarrobal inundable. 24-V-1994, *Mereles y Degen 5689* (FCQ!).

### ﻿Molecular phylogenetic analyses

A total of 21 terminal taxa were included, encompassing species previously assigned to the *E. grandiflorus* complex sensu Lehtonen and Falck (2011) and representatives from other sections of the genus. Five individuals of *E. nenufar* were sampled and sequenced de novo for all three loci. Sequence data for other taxa were obtained from published sources (Lehtonen and Myllys 2008; Lehtonen and Falck 2011). Three molecular markers were used, with the nuclear ITS having an aligned length of 702 bp, the nuclear *LEAFY* 260 bp, and the plastid *rpl22–rps19* 957 bp, resulting in a concatenated data matrix of 1919 characters.

**Figure 7.**
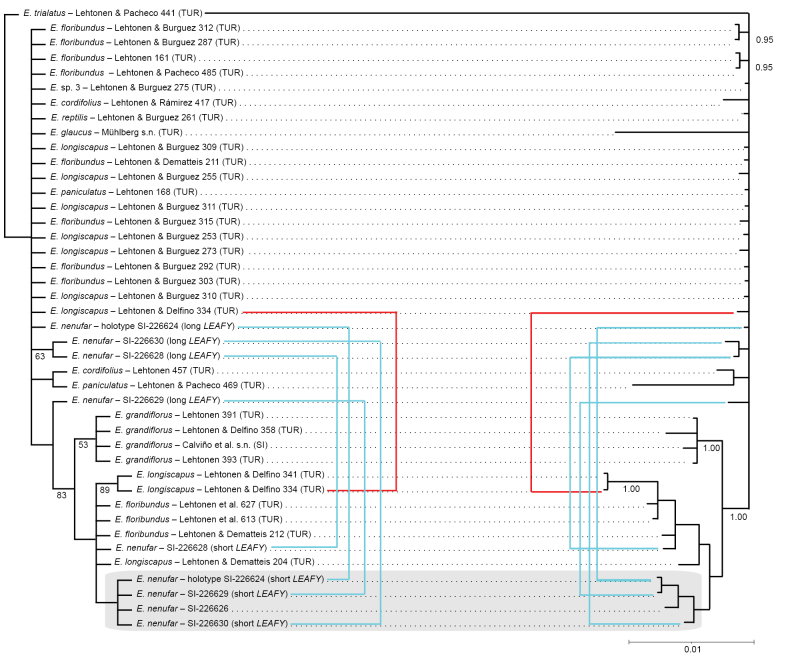
Phylogenetic placement of *Echinodorus nenufar* based on combined ITS, *LEAFY*, and plastid (*rpl22–rps19*) sequences. For heterozygous plants, the short *LEAFY* alleles were combined with ITS and plastid sequences, while the longer alleles were analyzed alone. Strict consensus tree from the parsimony analysis with jackknife support values ≥ 50 on the left and majority-rule Bayesian tree with posterior probabilities ≥ 0.95 on the right. Alleles phased separately from the same individuals are indicated by blue (*E. nenufar*) or red (*E. longiscapus*) connecting lines.

Phylogenetic trees inferred from the combined, phased dataset (ITS + *LEAFY* + plastid) remained poorly resolved. The parsimony analysis resulted in 5661 equally parsimonious trees with a length of 202 steps. Apart from *E. grandiflorus*, none of the species were resolved as monophyletic in the strict consensus tree or in the Bayesian majority-rule consensus. *Echinodorus nenufar* sequences concatenated to include the short *LEAFY* allele grouped together with some individuals of *E. longiscapus* and *E. floribundus* in a clade sister to *E. grandiflorus* (Fig. 7). The longer *LEAFY* allele was placed in a large unresolved polytomy containing morphologically diverse species, such as *E. paniculatus*, *E. glaucus*, *E. reptilis*, and *E. floribundus*, suggesting possible ancestral polymorphism or introgression. The results from parsimony and Bayesian analyses were congruent.

### ﻿Strict dichotomous key to *Echinodorus* (Chacoan Region, including Pampean and Uruguayense provinces)

**Table:** 

1	Fruiting heads (capitula) entirely enclosed by sepals, and leaf blades with pellucid markings forming a network independent of venation	***E. longipetalus***
–	Fruiting heads (capitula) not entirely enclosed by sepals, and leaf blades without a pellucid network independent of venation (i.e., no pellucid markings or only pellucid dots and/or lines)	**2**
2	Flowers 1–2 cm in diameter, stamens 13–15, and fruit with stylar beak 0.9–1.8 mm long	***E. berteroi***
–	Flowers 2.5–6 cm in diameter, stamens 15–60, and fruit with stylar beak 0.1–0.8 mm long	**3**
3	Leaf blades with a cordate base	**4**
–	Leaf blades with an attenuate base	**7**
4	Leaves lacking pellucid markings	***E. nenufar***
–	Leaves with pellucid markings present as dots and/or lines	**5**
5	Pellucid markings clearly present as both distinct dots and distinct lines; fruiting heads generally 1–3 cm in diameter	***E. longiscapus***
–	Pellucid markings not simultaneously present as both distinct dots and distinct lines (i.e., dots only, or dots with very faint lines, or variable dots without well-defined lines); fruiting heads generally outside the 1–3 cm diameter range	**6**
6	Leaves > 20 cm long; pellucid markings consisting of dots and distinct linear elements; plants > 100 cm tall	***E. grandiflorus***
–	Leaves ≤ 20 cm long; pellucid markings consisting of dots only; plants < 100 cm tall	***E. floribundus***
7	Achenes lacking glands; plants 40–200 cm tall with emergent leaves	***E. paniculatus***
–	Achenes with basal glands; plants < 40 cm tall, with usually submerged	**8**
8	Scapes with 3–6 whorls of flowers, each whorl bearing 4–10 flowers	***E. uruguayensis***
–	Scapes with 1–2 whorls, each whorl bearing 3(–5) flowers	**9**
9	Leaves linear to subulate, usually completely submerged; leaf margins entire; inflorescences erect, with numerous whorls along the rachis	***E. subalatus***
–	Leaves elliptic to lanceolate, usually at least partly emergent; leaf margins entire or slightly undulate; inflorescences decumbent, with few whorls along the rachis	***E. reptilis***

## ﻿Discussion

Christenhusz et al. (2018) erected a new genus, *Aquarius*, to accommodate most of the species in *Echinodorus*, including the *E. grandiflorus* complex, to which *E. nenufar* belongs. However, as discussed by Pestana et al. (2025), the description of *Aquarius* was ill-founded, and we reject it. The new species is therefore placed in *Echinodorus*, in accordance with the generally accepted taxonomic classification of Alismataceae (Lehtonen and Myllys 2008; Li et al. 2022; Pestana et al. 2025).

The taxonomy of the *Echinodorus grandiflorus* complex, to which *E. nenufar* clearly belongs, has long been problematic due to high morphological variability (Rataj 1969a, 1969b; Lehtonen 2018) and weak signal in phylogenetic analyses, even when species appear to differ ecologically (Lehtonen 2008). Even morphologically distinctive taxa, such as *E. paniculatus*, *E. cordifolius*, and *E. reptilis*, cannot be separated using the loci traditionally employed. Hybrids can be easily produced in *Echinodorus* and certainly occur in nature (Lehtonen and Falck 2011). Thus, the taxonomy of the *E. grandiflorus* complex remains unsatisfactory and will ultimately require genomic approaches to resolve.

*Echinodorus nenufar* can be consistently distinguished from all congeners by a unique combination of morphological characters. The most diagnostic feature is the corolla, with petals pale yellow to creamy white and a basal yellow blotch extending up to 30% of their length—contrasting sharply with the white petals of all other *Echinodorus*. Leaf morphology further supports its distinctness: mature individuals develop broadly cordate to suborbicular floating leaves, a form strikingly reminiscent of *Nymphaea*. This convergence with Nymphaeaceae is not merely visual but reflects functional adaptation to ephemerally flooded habitats.

Molecular results were limited in resolution but recovered *Echinodorus nenufar* as part of the *E. grandiflorus* species complex. The nuclear *LEAFY* marker revealed heterozygosity in most of the studied individuals, with two phased alleles displaying different affinities that may reflect ancestral polymorphism or introgression. Lack of phylogenetic signal and non-monophyly of species within the complex indicate recent and ongoing diversification. Although detailed ecological data are not available for comparative analyses, it seems evident that ecological specialization plays a role in diversification within the species complex. For example, *E. reptilis* is restricted to sandy riverbanks, and the largely sympatric species *E. longiscapus* and *E. grandiflorus* appear to occupy different niches (Lehtonen 2008). The unique ecological adaptations to ephemeral water bodies in the Chaco region underscore the evolutionary independence of *E. nenufar* within the species complex.

Conventional herbarium pressing often deforms or obscures taxonomically relevant traits, such as leaf outline, petiole anatomy, and delicate floral structures, especially in aquatic plants. The new protocols described here—combining antioxidant pretreatment, ventilated low-pressure pressing, and protective mounting—enable the preservation of entire aquatic specimens, including floating leaves, with minimal distortion. These techniques provide a replicable framework for improving the representation of aquatic taxa in herbaria.

In summary, recognition of *Echinodorus nenufar* is justified by a combination of diagnostic morphology and ecological specialization, while preliminary molecular evidence, although not conclusive, does not contradict this hypothesis. Its discovery enriches the diversity of *Echinodorus* in Argentina and Paraguay and highlights the evolutionary innovations of aquatic plants in ephemeral wetlands.

## Supplementary Material

XML Treatment for Echinodorus nenufar

## ﻿Additional information

### Use of AI

No use of AI was reported.

### Author ORCIDs

Pablo A. Calviño https://orcid.org/0000-0003-0944-599X

Samuli Lehtonen https://orcid.org/0000-0001-6235-2026

Marcos Waldbillig https://orcid.org/0009-0009-4942-9303

Tomás Acuña https://orcid.org/0009-0009-8211-4135

Felipe Alonso https://orcid.org/0000-0001-7227-2732

### Data availability

All of the data that support the findings of this study are available in the main text.

## ﻿Appendix 1

Specimens used in molecular analysis with GenBank accession numbers (*LEAFY*; ITS; *rpl22–rps19*). Newly generated sequences are shown **in bold**.

*Echinodorus cordifolius* (L.) Griseb.; Venezuela; Lehtonen 457 (TUR); EF088172; EF088078; PV549367. *E. cordifolius* (L.) Griseb.; Mexico; Lehtonen & Rámirez 417 (TUR); EF088173; EF088079; PV549366. *E. floribundus* (Seub.) Seub.; Bolivia; Lehtonen 161 (TUR); EF088153; EF088057; PV549374. *E. floribundus* (Seub.) Seub.; Venezuela; Lehtonen & Pacheco 485 (TUR); EF088175; EF088081; PV549365. *E. floribundus* (Seub.) Seub.; Brazil; Lehtonen et al. 613 (TUR); PV552002; PV534797; PV549354. *E. floribundus* (Seub.) Seub.; Brazil; Lehtonen et al. 627 (TUR); PV552001; PV534796; PV549353. *E. floribundus* (Seub.) Seub.; Argentina; Lehtonen & Dematteis 211 (TUR); **PV552007**; **PV534802**; PV549359. *E. floribundus* (Seub.) Seub.; Argentina; Lehtonen & Dematteis 212 (TUR); PV552008; PV534803; PV549360. *E. floribundus* (Seub.) Seub.; Paraguay; Lehtonen & Burguez 287 (TUR); PV552005; **PV534800**; PV549357. *E. floribundus* (Seub.) Seub.; Paraguay; Lehtonen & Burguez 292 (TUR); PV551998; PV534794; PV549350. *E. floribundus* (Seub.) Seub.; Paraguay; Lehtonen & Burguez 303 (TUR); **PV551997**; **PV534793**; **PV549349**. *E. floribundus* (Seub.) Seub.; Paraguay; Lehtonen & Burguez 312 (TUR); PV551995; PV534791; PV549347. *E. floribundus* (Seub.) Seub.; Paraguay; Lehtonen & Burguez 315 (TUR); PV552003; PV534798; **PV549355**. *E. glaucus* Rataj; Cultivated; Mühlberg s.n. (TUR); EF088178; EF088084; PV549363. *E. grandiflorus* (Cham. & Schltdl.) Mich.; cultivated; Lehtonen 393 (TUR); EF088164; EF088070; PV549368. *E. grandiflorus* (Cham. & Schltdl.) Mich.; Uruguay; Lehtonen & Delfino 358 (TUR); EF088180; EF088086; PV549361. *E. grandiflorus* (Cham. & Schltdl.) Mich.; Argentina; Lehtonen 391 (TUR); EF088160; EF088065; PV549343. *E. grandiflorus* (Cham. & Schltdl.) Mich.; Argentina; Calviño et al. s.n. (SI); **PV551994**; PV534789; **PV549345**. *E. longiscapus* Arechav.; Argentina; Lehtonen & Dematteis 204 (TUR); HM367245; EF088059; PV549373. *E. longiscapus* Arechav.; Paraguay; Lehtonen & Burguez 253 (TUR); **PV552000**; –; **PV549352**. *E. longiscapus* Arechav.; Paraguay; Lehtonen & Burguez 255 (TUR); PV552006; PV534801; PV549358. *E. longiscapus* Arechav.; Paraguay; Lehtonen & Burguez 273 (TUR); **PV551999**; **PV534795**; **PV549351**. *E. longiscapus* Arechav.; Paraguay; Lehtonen & Burguez 309 (TUR); EF088179; EF088085; PV549362. *E. longiscapus* Arechav.; Paraguay; Lehtonen & Burguez 310 (TUR); PV551996; PV534792; **PV549348**. *E. longiscapus* Arechav.; Paraguay; Lehtonen & Burguez 311 (TUR); **PV552004**; **PV534799**; **PV549356**. *E. longiscapus* Arechav.; Uruguay; Lehtonen & Delfino 334 (TUR); HM367247/HM367246; EF088068; PV549369. *E. longiscapus* Arechav.; Uruguay; Lehtonen & Delfino 341 (TUR); EF088158; EF088063; PV549370. *E. nenufar* Calviño et al. s.n.; Argentina; Calviño et al. s.n. (SI-226630); PV552009/**PV552010**; PV534790; **PV549346**. *E. nenufar* Calviño et al. s.n.; Argentina; Calviño et al. s.n. (SI-226626); **PV552011**; **PV534804**; **PV549375**. *E. nenufar* Calviño et al.; Argentina; Calviño et al. s.n. (SI-226629); PV552012/**PV552013**; PV534805; **PV549376**. *E. nenufar* Calviño et al s.n. .; Argentina; Calviño et al. s.n. (SI-226624); PV552016/**PV552017**; **PV534806**; **PV549377**. *E. nenufar* Calviño et al.; Argentina; Calviño et al. s.n. (SI-226628); **PV552014/PV552015**; PV534807; **PV549378**. *E. paniculatus* Mich.; Bolivia; Lehtonen 168 (TUR); EF088144; EF088048; PV549344. *E. paniculatus* Mich.; Venezuela; Lehtonen & Pacheco 469 (TUR); EF088176; EF088082; PV549364. *E. reptilis* Rataj; Paraguay; Lehtonen & Burguez 261 (TUR); EF088156; EF088061; PV549372. *Echinodorus* sp. 3; Paraguay; Lehtonen & Burguez 275 (TUR); EF088157; EF088062; PV549371. *E. trialatus* Fassett; Venezuela; Lehtonen & Pacheco 441 (TUR); EF088168; EF088074; PV549342.
